# Blockchain‐Empowered H‐CPS Architecture for Smart Agriculture

**DOI:** 10.1002/advs.202503102

**Published:** 2025-04-25

**Authors:** Xiaoding Wang, Qibin Wu, Haitao Zeng, Xu Yang, Hui Cui, Xun Yi, Md. Jalil Piran, Ming Luo, Youxiong Que

**Affiliations:** ^1^ National Key Laboratory for Tropical Crop Breeding Institute of Tropical Bioscience and Biotechnology Sanya Research Institute Chinese Academy of Tropical Agricultural Sciences Sanya Hainan 572024 China; ^2^ Fujian Provincial Key Lab of Network Security and Cryptology College of Computer and Cyber Security Fujian Normal University Fuzhou Fujian 350117 China; ^3^ College of Computer and Data Science Minjiang University Fuzhou Fujian 350108 China; ^4^ Department of Software Systems & Cybersecurity Monash University Melbourne VIC 3800 Australia; ^5^ School of Computing Technologies RMIT University Melbourne VIC 3000 Australia; ^6^ Department of Computer Science and Engineering Sejong University Seoul 05006 South Korea; ^7^ State Key Laboratory of Plant Diversity and Specialty Crops South China Botanical Garden Chinese Academy of Sciences Guangzhou 510650 China; ^8^ Key Laboratory of Sugarcane Biology and Genetic Breeding Ministry of Agriculture and Rural Affairs Fujian Agriculture and Forestry University Fuzhou Fujian 350002 China

**Keywords:** blockchain, human‐cyber‐physical systems, prospect, semantic blockchain, smart agriculture

## Abstract

This study integrates blockchain technology into smart agriculture to enhance its productivity and sustainability. By combining blockchain with remote sensing, artificial intelligence (AI), and the Internet of Things (IoT), a Human‐Cyber‐Physical System (H‐CPS) architecture tailored for agricultural applications is proposed. It supports real‐time crop management, data‐driven decision‐making, and transparent trading of agricultural products. A semantic‐based blockchain framework is introduced to address challenges in data management and AI model integration, optimizing production, improving traceability, reducing costs, and enhancing financial security. This framework directly addresses real‐world agricultural challenges, such as optimized irrigation, improved crop breeding efficiency, and enhanced supply chain transparency. These innovations provide practical solutions for modern agriculture, contributing to sustainable development and global food security. Further research and collaboration are encouraged to unlock its full potential in transforming agricultural practices.

## Graphical abstract

This article reviews the current system architectures for smart agriculture and proposes a blockchain‐empowered hierarchical cyber‐physical system (H‐CPS) framework. It discusses the challenges and outlines future research directions, including the use of semantic blockchain to handle complex data and models.

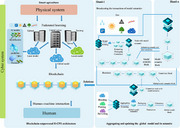

## 1 Introduction

Crops constitute the main source of nourishment globally, with the United Nations predicting that the world's population will reach 9.7 billion by 2050.^[^ ^1^ ^]^ Consequently, enhancing the agricultural output of crops and livestock is crucial to fulfill the continuous demand for food.^[^ ^2^ ^]^ Nonetheless, the Food and Agriculture Organization argues that merely increasing production is not required. Emphasizing the importance of making agricultural systems more sustainable and harnessing technology, research, and development is imperative for sustainable agriculture.^[^ ^3^ ^]^

**Figure 1:**
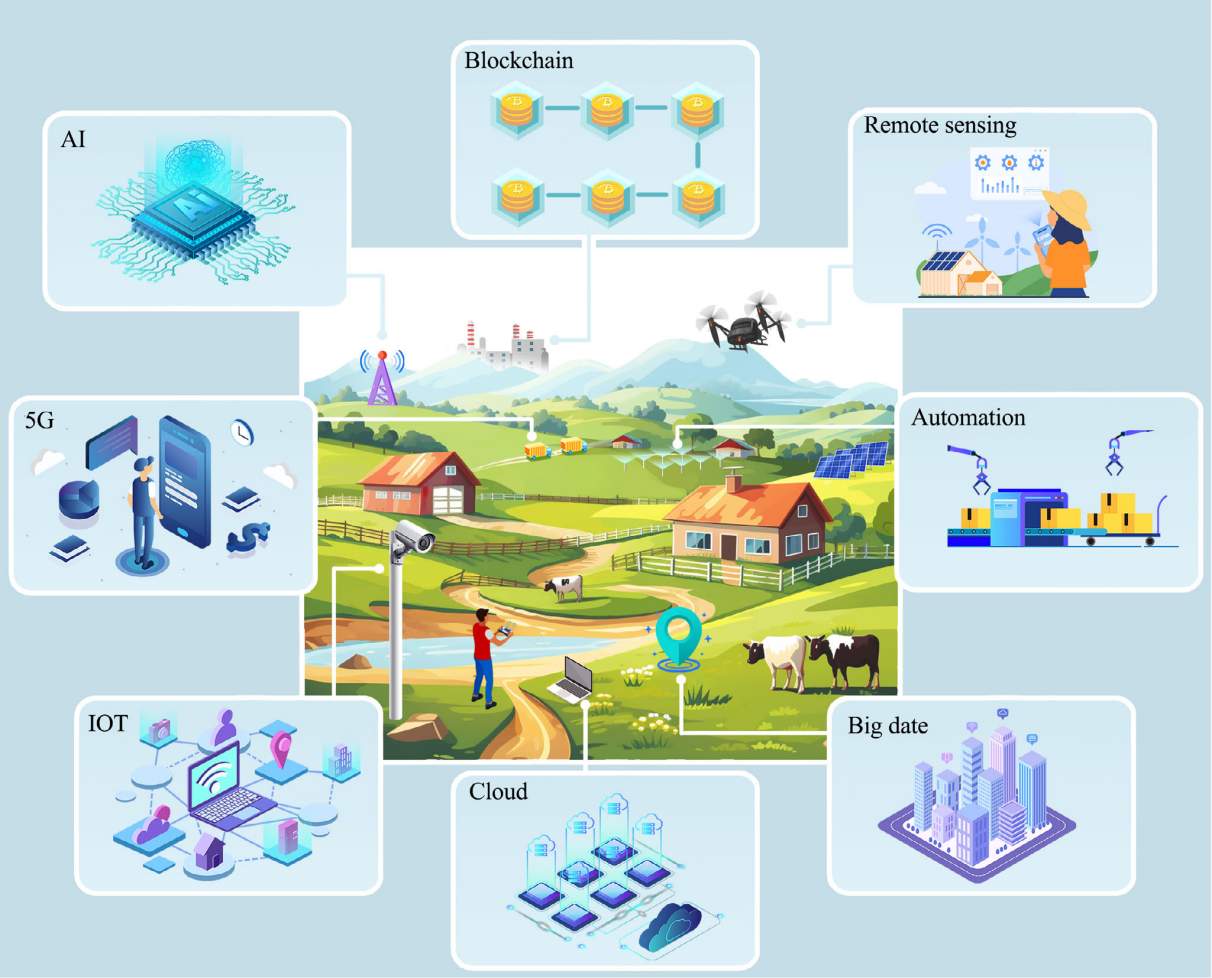
Smart agriculture integrates IoT devices (soil moisture sensors, drones), AI‐driven analytics (crop health prediction models), and blockchain‐based traceability (supply chain ledgers). The architecture enables real‐time data collection, automated decision‐making, and transparent transactions across stakeholders.

Industry 4.0, the Fourth Industrial Revolution, is a strategic effort to integrate disruptive digital technologies. It includes Internet of Things (IoT), blockchain, Artificial Intelligence (AI), cloud computing, big data analytics, augmented reality, digital twins, autonomous robotic systems, wireless sensor networks and Human‐Cyber‐Physical Systems (H‐CPS).^[^ ^4^ ^]^ This revolution significantly impacts various sectors, including agriculture, by promoting more intelligent, integrated, and data‐driven approaches. The shift has led to Agriculture 4.0 or smart agriculture (**Figure** **1**). It illustrates key components of smart agriculture, including IoT‐enabled soil sensors for precise irrigation, drones equipped with multispectral cameras for crop monitoring, and blockchain nodes that record supply chain data. These elements collectively enable dynamic resource optimization and secure data sharing. Essentially, smart agriculture uses information and technology to optimize agricultural systems.^[^ ^5^ ^]^ It offers farmers tools to tackle issues related to productivity, crop yield loss, and sustainability.^[^ ^6^ ^]^

The rising demand for high‐quality and abundant food is pushing the agricultural sector toward industrialization, necessitating a stronger supply chain.^[^ ^7^ ^]^ Blockchain technology plays a crucial role in documenting data across the entire process from seed to sale.^[^ ^8^ ^]^ Unlike conventional databases that use access control and encryption for security, blockchain ensures security through consensus protocols and its cryptographic nature.^[^ ^9^ ^]^ In agriculture, blockchain is mainly employed to ensure food safety by tracking production and storing specific data. However, agricultural progress is affected by numerous factors, such as climate change, economic cycles, and industry regulations.^[^ ^10^ ^]^ Consequently, traditional blockchain frameworks may fall short in fulfilling the requisites for sustainable agricultural growth. Addressing these issues, merging blockchain with emerging technologies like IoT, AI, and H‐CPS can improve several agricultural dimensions, including crop breeding, production decision‐making, product transactions, finance, quality and safety assurance, and sustainability.^[^ ^11^ ^]^

This study provides an overview of components in smart agriculture, emphasizing the role of blockchain role and its potential applications. The study offers these following contributions. First, it summarizes current smart agriculture architectures and their benefits. Second, it proposes a blockchain‐empowered H‐CPS architecture for smart agriculture 4.0, outlining hierarchical structures. Third, it presents concepts and applications of blockchain‐empowered H‐CPS in smart agriculture. Lastly, it addresses blockchain‐empowered H‐CPS challenges and future research, aiming to develop a semantic blockchain framework to manage large data volumes and complex models.

## 2 Current State of Smart Agriculture

Recently, smart agriculture has advanced through IoT technology, attracting considerable interest. Researchers have ingeniously employed IoT devices and networks to automate irrigation planning and improve agricultural data collection and monitoring. For instance, Sekaran et al. created an innovative IoT‐based agriculture framework using cloud computing to process real‐time crop sensor data, enabling automated decision‐making and reducing farmers' time and energy expenditure.^[^ ^12^ ^]^ Quy et al. reviewed IoT solutions by crafting an IoT ecosystem architecture, showing their incorporation into smart agriculture.^[^ ^13^ ^]^ Sushanth and Sujatha developed an intelligent agriculture system integrating IoT, wireless sensor networks, and cloud computing for automated irrigation and soil sensor data transmission.^[^ ^14^ ^]^

Integrating AI in smart agriculture can improve decision‐making, optimize resources, and boost crop yields. Chen et al. combined IoT with deep learning for a real‐time system identifying weather and pests in smart agriculture.^[^ ^15^ ^]^ Shukla et al. designed an IoT and machine learning‐based architecture that uses drone‐captured multispectral images to analyze crop health.^[^ ^16^ ^]^ Bu et al. developed an intelligent agriculture IoT system employing deep reinforcement learning, structured into four layers: data collection, edge computing, transmission, and cloud computing, enabling immediate decisions for optimized irrigation and better yields.^[^ ^17^ ^]^

Research efforts have also suggested novel smart agriculture solutions by merging IoT with blockchain technology. This fusion optimizes data handling throughout the agricultural value chain, ensuring a safe and open environment for transactions among stakeholders and promoting sustainable growth. Lin et al. unveiled a cutting‐edge agricultural framework incorporating blockchain and IoT. The main goal is to cultivate trust among participants by enabling agents to swiftly access agricultural data stored on the blockchain via smartphone applications. This system spans the entire process from cultivation to marketing. Chen et al. embedded a circular agriculture model within a blockchain infrastructure, leveraging smart devices and wireless sensor networks to autonomously gather and upload production and environmental data. This strategy enhances information parity, traceability for value chain members, and secures transactions.^[^ ^18^ ^]^ Recent trends emphasize the integration of AI and blockchain to advance intelligent agriculture. Frikha et al. proposed a solution combining deep learning, blockchain, and IoT for smart greenhouse management. This approach seeks to boost crop production while maintaining supply chain traceability.^[^ ^19^ ^]^

Despite the development of numerous smart agriculture systems, most of them fail to provide a cohesive framework design, thereby impeding seamless integration among systems. Sarkar et al. introduced the Cyber‐Physical Systems (CPS)‐based Agriculture System (CAS) framework.^[^ ^20^ ^]^ This leveraging the CPS framework, amalgamates sensing technologies, AI, and smart actuators, and categorizes the system into three parts: sensing, modeling, and actuation. The CAS architecture enhances efficiency, productivity, and sustainability in agriculture. Nonetheless, it might not directly address issues like user interaction and data privacy, as it predominantly concentrates on merging physical and network systems organically.^[^ ^21^ ^]^ Machine‐human interaction is an essential component in agriculture, making the H‐CPS concept promising for advancing intelligent agriculture and establishing a comprehensive framework for pioneering agricultural systems.^[^ ^22^ ^]^

## 3 Human‐Cyber‐Physical Systems

H‐CPS is structured into three tiers: human, network, and physical systems.^[^ ^23^ ^]^ The hallmark of H‐CPS is the synergistic collaboration between machines and humans in operational settings. The design purports to integrate with, rather than replace, human expertise to boost productivity and performance.^[^ ^24^ ^]^ Within an H‐CPS, the physical system provides data and executes operations, the network system handles data processing and analysis, while humans contribute expertise and make decisions.^[^ ^25^ ^]^ The initial H‐CPS setup primarily included machines for data collection and automated operations, with the Industrial IoT and cloud platforms facilitating integration. The human‐input knowledge base was crucial.^[^ ^26^ ^]^ Recently, H‐CPS has incorporated innovations like blockchain and AI. Blockchain aids system integration, whereas AI supports self‐learning, analysis, and cognition.^[^ ^27^ ^]^ Consequently, the H‐CPS knowledge base now results from contributions from self‐learning cognitive modules in both humans and network systems, including tacit knowledge difficult for humans to express.^[^ ^28^ ^]^ In H‐CPS, humans function as creators, managers, and operators whose skills and intellectual capabilities are enhanced, thereby optimizing productivity.^[^ ^29^ ^]^

## 4 Blockchain‐Empowered H‐CPS Architecture for Smart Agriculture

In the Industry 4.0 era, H‐CPS is applied in diverse areas such as smart manufacturing, intelligent buildings,^[^ ^30^ ^]^ smart grids,^[^ ^31^ ^]^ autonomous vehicles,^[^ ^32^ ^]^ advanced nuclear power,^[^ ^33^ ^]^ and intelligent transportation.^[^ ^34^ ^]^ These applications aid in accumulating, using, transmitting, and inheriting agricultural knowledge, thus boosting the ability of agricultural systems to tackle complex problems, which enhances system modeling and decision‐making.

H‐CPS can be implemented as either a single system, like smart driving vehicles, or as a distributed system connected by a communication network.^[^ ^35^ ^]^ Blockchain, a prime example of distributed systems,^[^ ^36^ ^]^ has great potential to improve H‐CPS performance and reliability. It provides decentralized, tamper‐resistant data storage and a mechanism for verifying interactions, thus fostering a trusted network environment. Thanks to blockchain's decentralized nature, H‐CPS can more readily adapt to diverse environments and requirements, without centralized management.^[^ ^37^ ^]^ Additionally, blockchain integration can boost the traceability of agricultural products and data.^[^ ^38^ ^]^ Combined with AI and remote sensing, H‐CPS can establish a secure, intelligent, traceable, and interactive real‐time agricultural system linking humans to the physical network.

**Figure 2:**
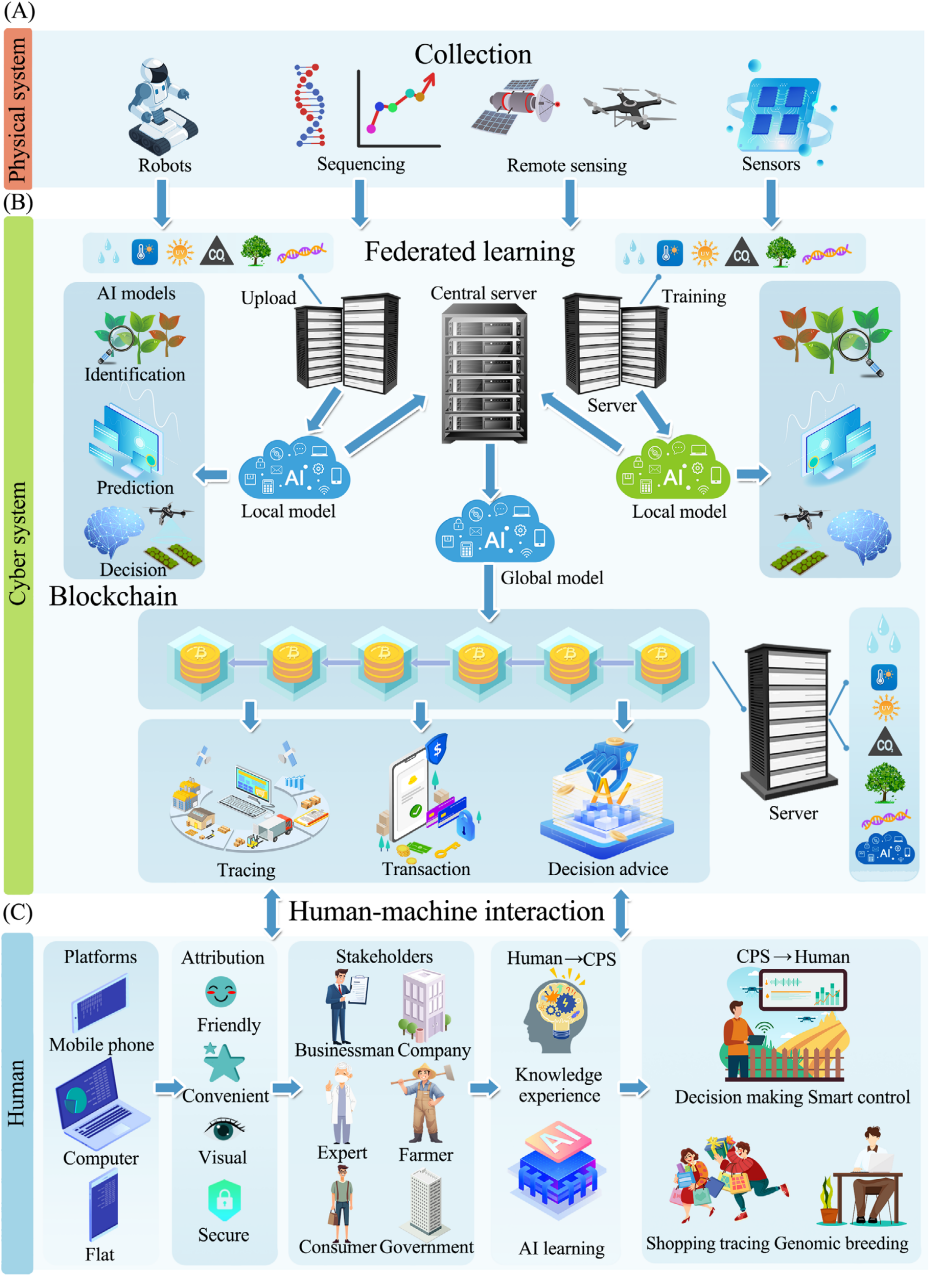
Blockchain‐empowered H‐CPS architecture for smart agriculture.

We introduce here a smart agriculture system architecture powered by blockchain technology (**Figure** **2**). This architecture follows the H‐CPS design framework, which is structured into three layers: the physical system layer, the network system layer, and the human‐machine interaction layer. The blockchain serves as a flexible, comprehensive human‐machine interaction platform. Within the physical system layer, sensors gather specific information from the environment or crops. This data can be preprocessed with edge computing technology on‐site or kept as raw data.^[^ ^39^ ^]^ Subsequently, the data are sent to the network system layer using communication methods such as 5th‐Generation Mobile Communication Technology (5G),^[^ ^40^ ^]^ WIreless Fidelity (WIFI),^[^ ^41^ ^]^ and Long Range Radio (LoRa).^[^ ^42^ ^]^ Within this layer, AI models are utilized to extract actionable insights and build inference models from the data.^[^ ^43^ ^]^ Considering data differences across various regions and crop types, a model effective in one area may not be applicable elsewhere.^[^ ^44^ ^]^ Therefore, it is necessary to train a comprehensive global model, with smart contracts on the blockchain facilitating the deployment of these AI models.^[^ ^45^ ^]^ Conveniently, the network system layer provides precise information to support stakeholders in agricultural decision‐making processes.^[^ ^46^ ^]^ Stakeholders engage with the network system via a blockchain platform, thereby initiating agricultural production activities within the physical system layer. Moreover, different data streams, like order details, raw material information, and pricing data, are securely stored on the blockchain, with operations time‐stamped to safeguard data privacy.^[^ ^47^ ^]^ This system enables agricultural producers to engage in secure blockchain‐based commodity transactions, while allowing consumers to verify the origin of agricultural products throughout the supply chain, bolstering trust on both sides.

### 4.1 The Physical System Layer

This section explores the physical system domain within the blockchain‐enabled H‐CPS architecture for smart agriculture. It outlines various types of agricultural sensors, robots, drones, satellite remote sensing technologies, and high‐throughput sequencing technologies used for crop breeding.

In smart agriculture, sensing devices are utilized through both proximity sensors and remote sensing methods. These sensors are pivotal for real‐time data gathering in agricultural fields, enabling farmers to continuously track variables like humidity, temperature, soil, water, and crop quality. **Table** **1** outlines the main sensor categories, their measurement parameters, and application examples. When combined with blockchain technology, they enhance the creation of digital information assets.^[^ ^48^ ^]^ According to conditions specified in smart contracts, blockchain allows sensors to initiate response actions.^[^ ^49^ ^]^ Besides stationary sensors, sensor‐equipped robots can function as mobile proximity sensing platforms for crop and environmental observation, indicating their capability as task performers.^[^ ^50^ ^]^ For instance, small, specialized robots have been tasked with scouting and weeding,^[^ ^51^ ^]^ while commercial robots are predominantly applied for seeding, planting, harvesting, and pesticide dispersion.^[^ ^52^ ^]^ Proximity sensing occurs when sensors are in contact with or near the target objects. Conversely, remote sensing involves sensors on carrying platforms, such as airborne or orbiting types, with drones being a classic example of airborne platforms and satellites as orbiting ones.^[^ ^53^ ^]^ Remote sensing periodically captures detailed data over expansive areas using varied spectral wavelengths from afar, generating time‐series data crucial for assessing dynamic changes within that area,^[^ ^54^ ^]^ supplying an effective tool for monitoring crop growth, crop mapping, and yield forecasting.^[^ ^55^ ^]^ Since the 1970s, satellites have been an agricultural asset for detecting crop diseases and water stress.^[^ ^56^ ^]^ However, constraints like high expenses, limited imaging capacity, and a lack of automated image analysis frameworks hinder its broader agricultural use, particularly in small‐scale farming.^[^ ^57^ ^]^ Consequently, drones have gained popularity as a more adaptable, cheaper alternative for small farms. They equipped with pesticides, multispectral cameras, and hyperspectral imagers facilitate precision agriculture tasks like pesticide application, weed detection, and disease diagnosis.^[^ ^58^ ^]^ Sensor technology, along with its platforms, enables the collection of environmental and crop phenotypic data.

**Table 1:** Different sensor categories, measurement targets, and applications used in smart agriculture.

Sensing directions	Sensed target attributes	Sensors	Agricultural management applications
Acoustics	Amplitude, Phase, Polarization, Spectrum, Wave velocity	Ultrasonic distance sensor	Wind speed,^[^ ^61^ ^]^ Hardwood borer detection,^[^ ^62^ ^]^ Crop canopy height estimation^[^ ^63^ ^]^
Biology	Density, Biomass, Chlorophyll concentration, Species type	RGB camera, Near‐infrared (NIR) sensor, Multispectral sensor	Plant weight measurement,^[^ ^64^ ^]^ Biomass^[^ ^65^ ^]^
Chemistry	pH, Air quality, Gas type	Gas sensor, pH sensor	Air quality,^[^ ^66^ ^]^ Irrigation water pH, Greenhouse CO2,^[^ ^67^ ^]^ Soil pH value^[^ ^67^ ^]^
Electrics	Charge, Current, Resistance, Conductivity, Voltage	Soil moisture sensor, (capacitive or resistive) humidity sensor	Soil moisture content,^[^ ^65^ ^]^ Air humidity,^[^ ^66^ ^]^ Soil electrical conductivity,^[^ ^67^ ^]^ Stomatal conductance, Liquid flow estimation
Machine learning	Position, Mass, Flow rate, Shape	Pressure sensor, Strain gauge‐based weighing sensor	Continuous plant weight,^[^ ^64^ ^]^ Air pressure,^[^ ^66^ ^]^ Stem growth, Wind speed, Fruit growth measurements
Optics	Amplitude, Phase, Spectrum, intensity	Fluorescence sensor, Multispectral sensor, Hyperspectral sensor	Canopy temperature,^[^ ^66^ ^]^ Leaf disease detection,^[^ ^68^ ^]^ Changes in light intensity of crop canopy,^[^ ^69^ ^]^ Chlorophyll types
Thermal	Temperature, Flux, Specific heat	Temperature sensor	Estimation of soil moisture content^[^ ^70^ ^]^

Furthermore, gathering heritage data is vital for advancing breeding efficiency. High‐throughput sequencing can concurrently identify the sequences of various DNA or RNA fragments in a highly parallel fashion.^[^ ^59^ ^]^ This feature enables comprehensive analysis of certain genes, proteins, and their roles in related crops, eventually pinpointing critical genes and regulatory components, thus improving breeding efficiency.^[^ ^60^ ^]^

Breakthroughs in next‐generation sensors, remote sensing, robotics, and high‐throughput sequencing are crucial in swiftly acquiring large‐scale crop phenotype, genotype, and environmental data.^[^ ^71^ ^]^ The physical system layer will soon autonomously collect extensive plant data through various sensing modes across multiple locations and environments.^[^ ^72^ ^]^ This vast data can be stored on the blockchain for traceability or integrated seamlessly to train AI models, aiding informed decision‐making.

### 4.2 The Network System Layer

Optimizing agricultural production management and making informed crop breeding decisions rely heavily on a robust framework for data storage, computational modeling, and analytical reasoning. This section focuses on the network system layer of the smart agriculture H‐CPS architecture, which is enhanced with blockchain technology. It presents an overview of AI and blockchain concepts, examines their applications in agriculture, and evaluates how the network system layer supports data storage, computational modeling, and analytical reasoning. Agriculture is thus progressively incorporating AI to aid farmers with tasks such as crop selection, soil management, pest and disease control, yield estimation, and price prediction, thus boosting overall productivity.^[^ ^73^ ^]^ The proposed architecture employs several methods:

1. Deep Learning. This methodology mimics the human brain by forming multi‐layered neural network models to analyze data and recognize patterns. It is used in tasks such as classification, prediction, and generation. Deep learning uncovers complex features via training on large datasets.^[^ ^74^ ^]^ Digital remote sensing data become easier to manage and interpret, facilitating deeper analyses for automatic crop, weed, and plant disease identification, classification,^[^ ^75^ ^]^ and crop yield estimation.^[^ ^76^ ^]^ It aids in developing breeding models that assess genotype data, predict genetic traits, and accelerate crop improvement.^[^ ^77^ ^]^
2. Deep Reinforcement Learning. This approach employs deep neural networks to train intelligent agents for task execution in complex settings, optimizing decisions by maximizing cumulative rewards.^[^ ^78^ ^]^ It allows smart farmers to autonomously understand and make decisions,^[^ ^79^ ^]^ significantly boosting the efficiency and quality of agricultural production. Applications encompass robot and drone path planning,^[^ ^80^ ^]^ intelligent irrigation control,^[^ ^81^ ^]^ and crop growth monitoring.^[^ ^82^ ^]^ Furthermore, it aids in effective decision‐making for production and storage in blockchain‐based supply chains, improving management ease.^[^ ^83^ ^]^
3. Federated Learning. This is a decentralized learning approach where model training occurs across various local devices or servers rather than on a single central server. Data remains on local devices, with only the trained model parameters communicated to the central server for aggregated updates, thereby enhancing data privacy.^[^ ^84^ ^]^ In the context of smart agriculture, federated learning facilitates AI model training collaboratively without exchanging sensitive information among farmers, researchers, and organizations.^[^ ^85^ ^]^ Rather than data, stakeholders share model updates, combining them into a global model that incorporates knowledge from diverse regions for purposes such as detecting pests and diseases, yield forecasting, and crop breeding. This model accommodates variations in weather, soil conditions, and offers broader applicability.

**Figure 3:**
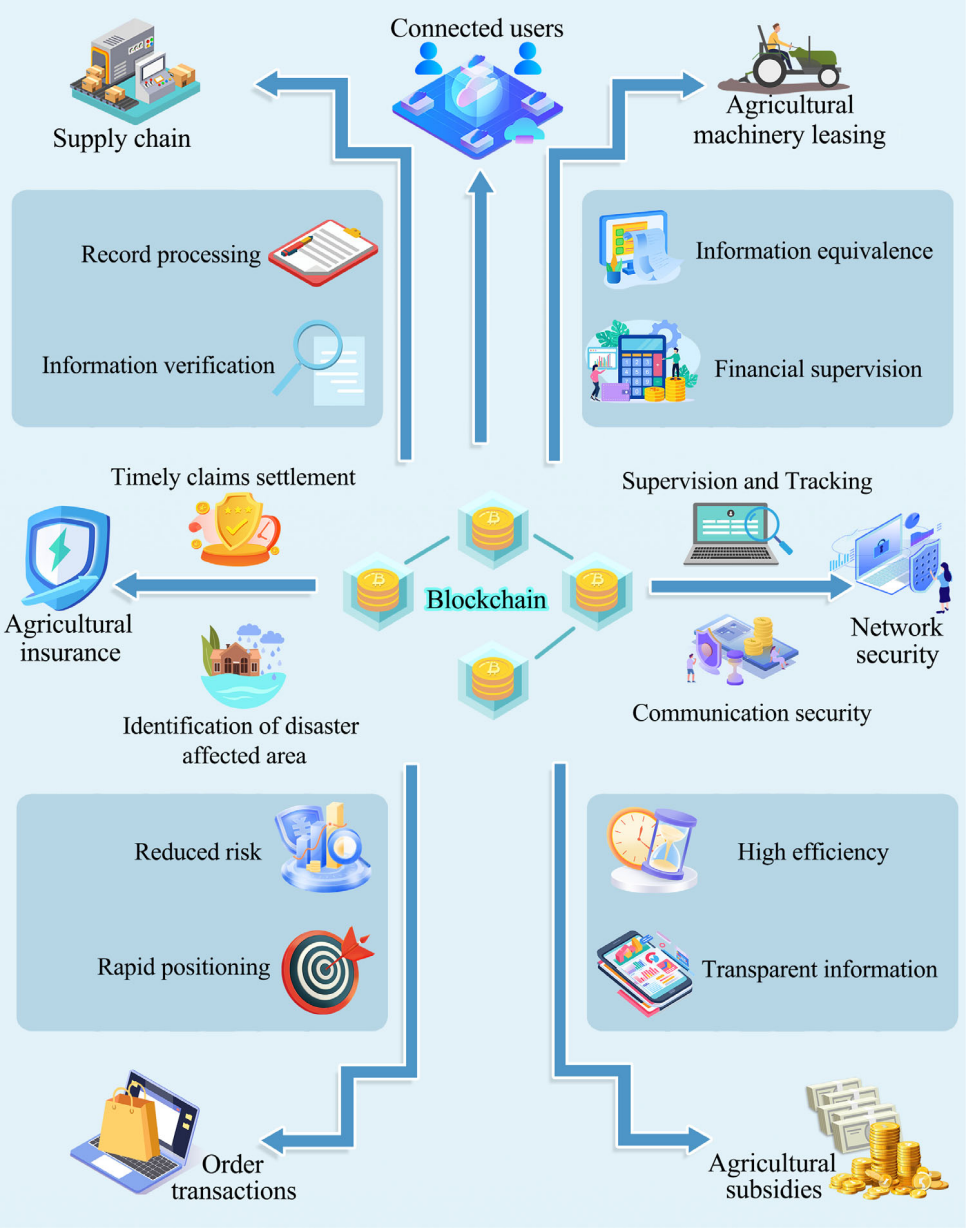
Blockchain applications in smart agriculture: 1) Supply chain traceability—farm‐to‐table data (e.g., seed origin, pesticide logs) is hashed and stored on‐chain; 2) IoT security—device authentication via smart contracts; 3) Insurance automation—weather data triggers payout via oracles; 4) Subsidy distribution—eligibility checks through consensus; 5) Machinery leasing—ownership records updated via decentralized ledgers; 6) Transaction integrity—timestamped orders prevent fraud.

Storing agricultural data securely is crucial for intelligent farming systems. Data at the physical system layer, handled by supply chain participants like producers, wholesalers, and retailers across different regions, faces risks of tampering and attacks in conventional database systems.^[^ ^86^ ^]^ Blockchain has emerged as a solution in smart agriculture to tackle these challenges. Each node can propose new transactions, but they must adhere to specific protocols for node acceptance or block inclusion. A block, which includes transaction records and a unique encrypted hash, connects to the preceding block, ensuring tamper resistance and data security once it is added to the chain.^[^ ^87^ ^]^ Smart contracts enhance blockchain efficiency and security, serving as programs executed on the blockchain to implement contract logic.^[^ ^88^ ^]^ These contracts automate code‐based contract management, reducing dependence on centralized authorities. Blockchain integration into smart agriculture improves transparency, traceability, and tamper resistance, promoting smart agriculture and advancing industry digitization.^[^ ^89^ ^]^ **Figure** **3** illustrates blockchain's varied applications in smart agriculture.

In Smart Agriculture, blockchain encompasses several domains, including the supply chain, IoT network security, transaction processing, subsidies, insurance, and machinery leasing. For instance, in blockchain‐enabled supply chains (Figure 3), farmers upload crop data (genetic traits, fertilizer usage) to an immutable ledger. Smart contracts automatically validate compliance with organic certification standards, while consumers trace product origins via Quick Response (QR) codes linked to blockchain records.

1. Supply Chain. Blockchain provides a secure, unchangeable record of the complete journey of agricultural goods from their production to the point of sale, thus allowing consumers to trace the origins effortlessly. It also archives essential data from the initial supply chain stages, like seed quality, genetic traits, fertilizers, and pesticides.^[^ ^90^ ^]^ The transparency allows all supply chain participants to verify the data, boosting consumer trust, ensuring food safety, and minimizing fraud.
2. IoT Network Security. In precision agriculture, blockchain protocol manages the transactional data exchanges between physical devices such as sensors on a decentralized ledger, thus enabling secure supervision and tracking.^[^ ^91^ ^]^ Additionally, it sets communication guidelines among sensors, enhancing secure communication and bypassing the complex procedures of Public Key Infrastructure (PKI) certificate swaps seen in Transport Layer Security (TLS).^[^ ^92^ ^]^
3. Order Transactions. Blockchain platforms surpass traditional e‐commerce systems for agricultural products by minimizing information tampering risks. In dispute scenarios post‐transaction, blockchain swiftly identifies the contentious link, thereby aiding traceability.^[^ ^93^ ^]^ Moreover, smart contracts streamline transaction processes, cut down costs, and employ decentralized structures to bolster system resilience.
4. Agricultural Subsidies. Blockchain optimizes policies supporting agricultural progress and cutting production costs. Conventional subsidy programs struggle with opaque information and lagging updates.^[^ ^94^ ^]^ A blockchain‐powered real‐time database allows transparent, efficient subsidy management. Furthermore, smart contracts use gathered farmer eligibility data to meet subsidy prerequisites, and when conditions are met, they automatically dispense funds to farmers promptly and accurately.^[^ ^95^ ^]^
5. Agricultural Insurance. Blockchain pulls data from weather databases and aligns with land records. This enables automatic disaster area identification without manual intervention, streamlining insurance payouts directly to farmers' wallets and boosting claim efficiency. Additionally, to counter employees inflating premiums for personal gain or farmers falsifying data, blockchain performs unbiased assessments using logged data, fostering trust between insurers and farmers.
6. Machinery Leasing. This approach aids farmers in obtaining farming equipment.^[^ ^96^ ^]^ Currently, the model faces hurdles like mitigating leasing risks and enhancing management effectiveness. Blockchain's decentralized trust model makes the machinery leasing landscape more transparent, effectively tackling issues such as information asymmetry, high negotiation costs, and fund management. It also helps financial bodies address transaction genuineness concerns.^[^ ^97^ ^]^

To sum up, the combination of blockchain and AI technology enables data analysis and predictive modeling to boost supply chain efficiency, refine production and sales strategies, cut costs, and enhance profits within the network system layer.^[^ ^98^ ^]^ When paired with deep and reinforcement learning, storing information such as weather patterns, data gathered from the physical system layer, and crop market prices on the blockchain can guide farmers in making well‐informed decisions about when to plant or sell specific crops.^[^ ^99^ ^]^ Using federated learning, farmers can also conduct precise irrigation, oversee pest and disease management, and forecast crop growth based on accurate data derived from blockchain and decision‐making models.^[^ ^100^ ^]^

### 4.3 The Human‐Machine Interaction Layer

The human‐machine interaction layer is vital for the collaboration between the agricultural system and human users in the blockchain‐enabled H‐CPS architecture for smart agriculture. In this section, we explore critical factors across the human‐machine interaction layer, such as real‐time data visualization, security assurance, and user‐friendliness, which seamlessly enhance interaction between the agricultural system and stakeholders, advancing the development of intelligent agriculture.

In H‐CPS, the physical system layer collects data through sensing technologies, while the network system layer is responsible for data transmission, storage, and processing. As an integral part of the system, humans connect with the entire system through interaction and decision‐making, allowing H‐CPS to coexist and symbolize between humans and machines, ultimately leading to efficient and effective production.^[^ ^101^ ^]^ In the context of Industry 4.0, the inseparable integration of humans and technology has given rise to human‐centered systems.^[^ ^102^ ^]^ In system operation, common human‐related issues are fatigue, operator errors, and lack of motivation, which can be addressed using machine assistance. However, specific human skills such as system design, root cause analysis, and fault diagnosis are challenging for machines to replace.^[^ ^103^ ^]^ In the collaborative interaction between humans and intelligent systems, advanced technologies enhance human skills and create more interfaces for interaction with machines, thereby maximizing the efficiency of collaboration within the system. In H‐CPS decision‐making, machine learning models can analyze data at the lowest granularity, while humans excel at extracting knowledge derived from decision‐making. Therefore, humans can transfer their expertise, skills, knowledge, and intelligence to machines through expert system training and AI technologies. As a result, AI agents must acquire knowledge through task execution and interact with humans to make the system learn human behavior and support human decision‐making, which means receiving assessment feedback from human observers is crucial in H‐CPS systems.^[^ ^104^ ^]^

**Figure 4:**
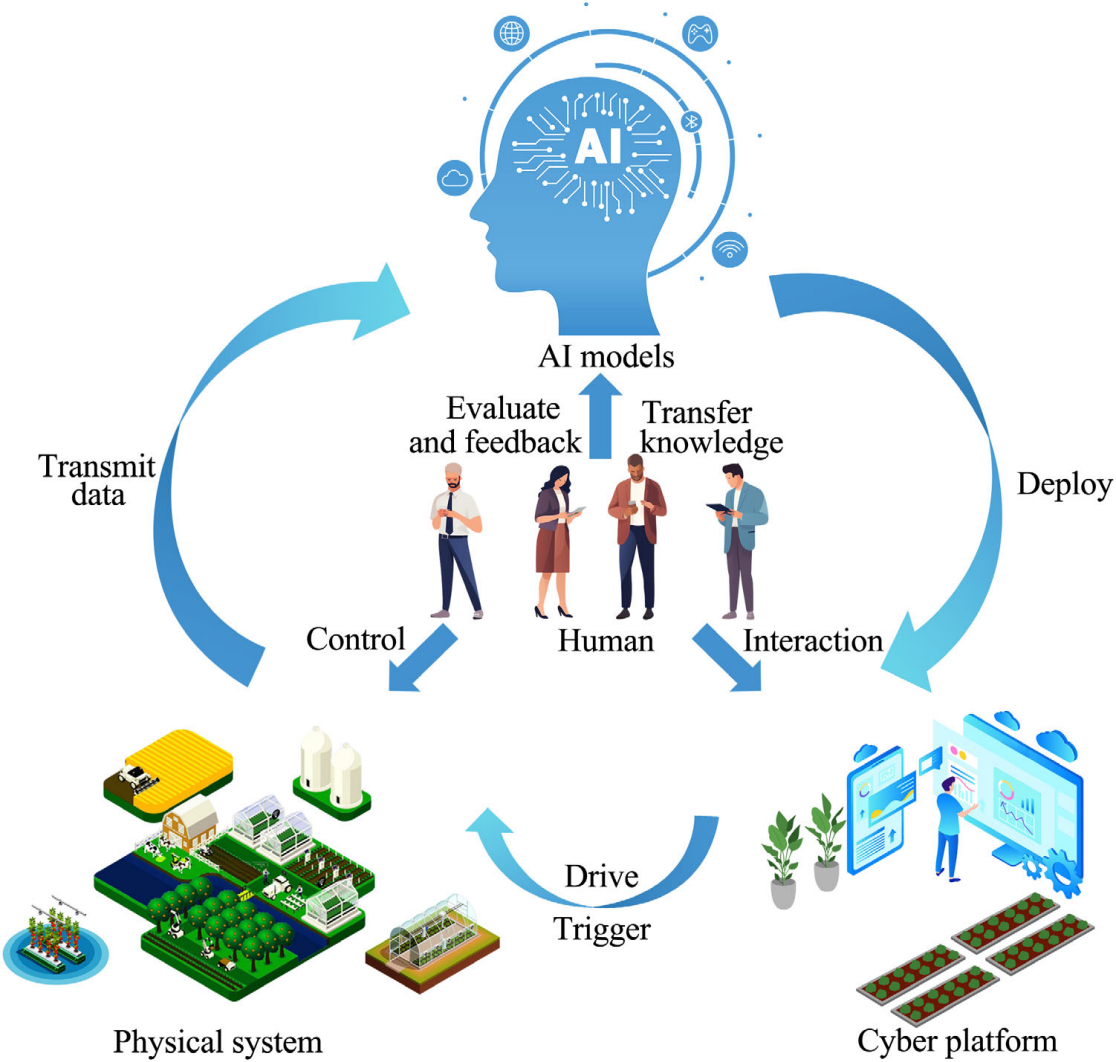
A typical scenario of human‐machine interaction in the blockchain‐empowered H‐CPS architecture for smart agriculture.

In the intelligent agricultural system architecture based on H‐CPS, the human‐machine interaction layer is manifested in three aspects. First, humans transfer cognitive and learning capabilities to the network system. Second, the network system considers user interaction, data privacy, and data operability related to humans to adapt to the agricultural domain. This approach necessitates the initial design of a user‐friendly interface, providing an intuitive graphical user interface and easy‐to‐understand controls. A visual data platform is also needed to provide users with real‐time information on fields, crops, and the status of orders. At the same time, the security of sensitive data and transactions is also crucial to prevent unauthorized access. Blockchain platforms, such as Ethereum and Hyperledger Fabric,^[^ ^105^ ^]^ can serve as user operation platforms, facilitating data management and ensuring data security.^[^ ^106^ ^]^ The blockchain online designed for users provides a standard view of all functional modules. Third, personalized recommendations are an important aspect of a human‐centered approach. By offering personalized agricultural advice through human‐machine dialogue, users receive customized recommendations optimized according to their specific needs and field conditions (**Figure** **4**).^[^ ^107^ ^]^

## 5 Discussion

Conventional H‐CPS structures in agriculture typically adhere to a three‐tier model. This includes a physical layer dedicated to real‐time data gathering through sensors and drones, a network layer focused on centralized data transmission and analysis using IoT and cloud computing, and a human‐machine interaction layer for user management and decision‐making.^[^ ^108^ ^]^ While these systems have improved automation and resource management, they continue to face challenges such as limited scalability, inadequate data security, restricted interoperability, and insufficient supply chain transparency. Furthermore, their reliance on human involvement reduces efficiency and responsiveness.^[^ ^109^ ^]^

Not surprising is that blockchain can empower H‐CPS through decentralized trust via consensus protocols (e.g., Proof‐of‐Authority), enabling secure, intermediary‐free transaction validation and data integrity in distributed agricultural networks. Smart contracts automate operations like irrigation or subsidies when sensor thresholds (e.g., soil moisture <30%) are met, enhancing efficiency. Cross‐chain protocols ensure semantic interoperability by integrating heterogeneous data (e.g., satellite imagery with IoT sensors) for unified analytics. This framework can reduce centralized dependency, mitigate systemic risks, and ensure sustainability compliance, fostering resilient and transparent agricultural ecosystems.

According to above analysis, a blockchain‐empowered H‐CPS framework has been proposed, integrating blockchain, IoT, AI, and federated learning into a decentralized, transparent, and intelligent agricultural network. It enhances the traditional three‐tier model by introducing edge computing at the physical layer, allowing data to be preprocessed as it is collected, thereby minimizing transmission delays. At the network layer, centralized storage systems are replaced with blockchain‐enabled distributed ledgers, ensuring secure and tamper‐resistant data management while facilitating automated processing through smart contracts. The interaction layer leverages blockchain platforms for real‐time monitoring and secure data sharing, enhanced by AI‐driven predictive models that provide personalized decision‐making support for users. This advanced architecture not only addresses the limitations of traditional H‐CPS frameworks in scalability, security, and transparency but also introduces innovative solutions to improve operational efficiency and decision‐making. When compared to conventional frameworks, the practical benefits of blockchain‐based H‐CPS systems are evident. The decentralized nature of blockchain reduces reliance on centralized storage, thereby enhancing data security and resilience against faults. Immutable ledgers ensure transparency throughout the agricultural supply chain, enabling traceability across every phase—from planting to processing and distribution.^[^ ^110^ ^]^ Automated smart contracts streamline tasks such as irrigation and fertilization using real‐time data, significantly reducing manual intervention and improving operational efficiency.^[^ ^111^ ^]^ Additionally, integrating AI and federated learning allows the system to utilize both global and regional datasets securely, enabling real‐time predictive analytics and precision management while minimizing human involvement. These innovations collectively provide a robust solution to the complex challenges of modern agriculture, including scalability, transparency, and responsiveness.^[^ ^112^ ^]^

**Figure 5:**
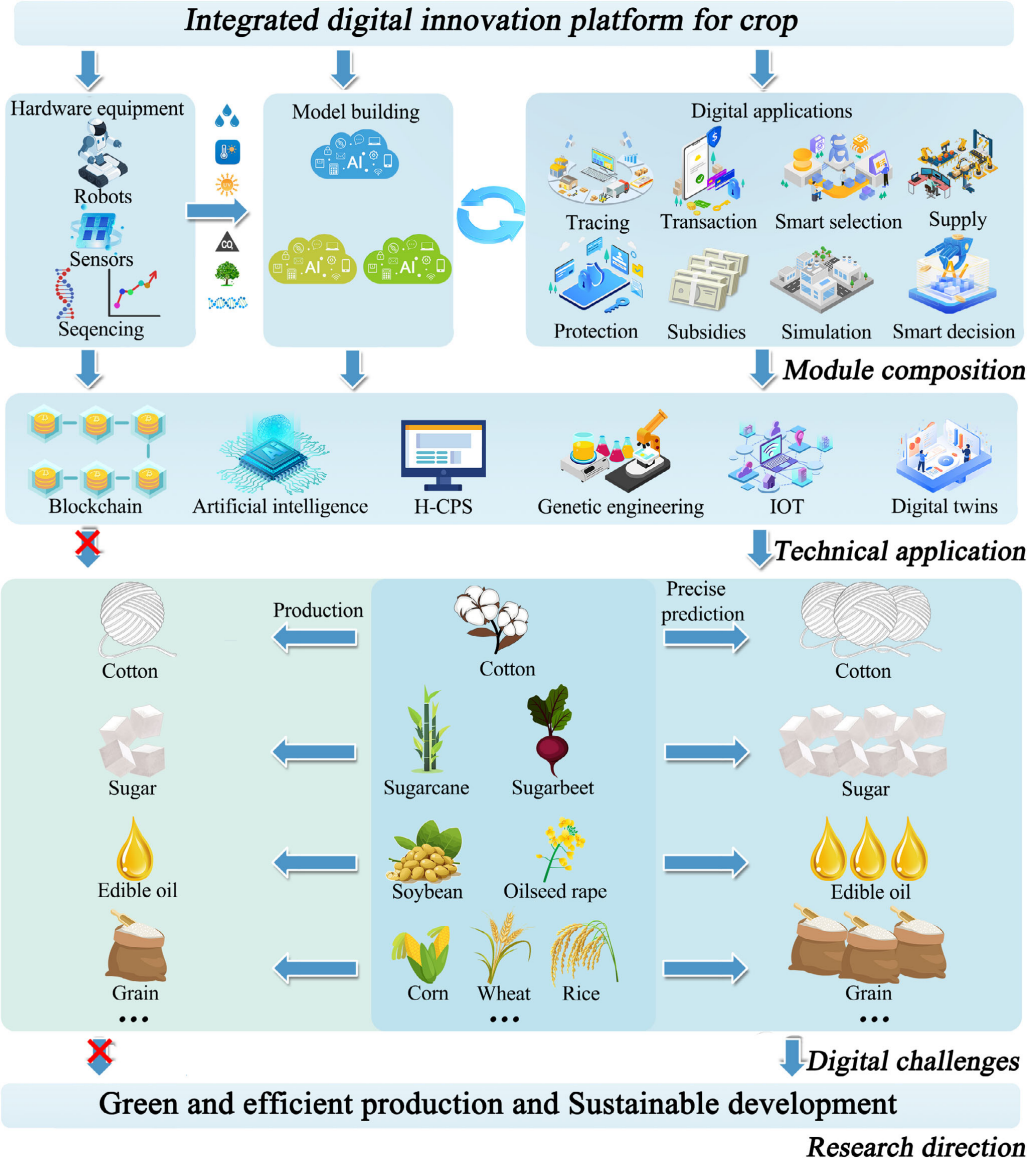
A digital crop pilot project based on H‐CPS, blockchain and AI.

Several pilot studies have demonstrated the efficacy of blockchain‐based H‐CPS architectures in real‐world agricultural applications.^[^ ^113^, ^114^, ^115^ ^]^ For instance, the Institute of Tropical Bioscience and Biotechnology of the Chinese Academy of Tropical Agricultural Sciences, in collaboration with its partners, has implemented a digital sugarcane pilot project in key planting regions such as Guangxi and Hainan provinces. This initiative integrates H‐CPS, blockchain, AI, IoT, and big data technologies to address critical challenges in sugarcane cultivation, production, and management, while promoting the digitalization and intelligent transformation of the industry. Building on this success, similar pilot projects have been launched for staple crops and strategic resources such as rice, wheat, maize, cotton, soybeans, and rapeseed. For example, in rice‐growing areas like Jiangsu and Hunan provinces, the integration of H‐CPS and blockchain has improved irrigation efficiency, pest control, and yield predictions. Likewise, in wheat and maize regions such as Inner Mongolia and Heilongjiang, AI and IoT technologies have optimized fertilizer use and pest management, leading to higher yields and more sustainable practices.^[^ ^116^ ^]^ The extension of these technologies to crops like cotton, soybeans, and rapeseed highlights the broad applicability of the digital architecture to diverse agricultural contexts (**Figure** **5**).

The adoption of AI‐driven systems across these projects provides significant benefits by enhancing precision and decision‐making capabilities. For example, AI algorithms analyze environmental data, soil nutrients, and crop‐specific physiological indicators to generate actionable insights, such as optimizing irrigation schedules or predicting pest outbreaks. These insights enable farmers to make data‐driven decisions, improving both crop yield and resource efficiency. Besides, AI algorithms are also expected to improve crop yield and quality by up to 20%, applicable to a wide variety of crops. Meanwhile, integrated smart water‐fertilizer‐pesticide systems, informed by AI analytics, can enhance resource utilization efficiency by 30%, reducing waste. Furthermore, drone‐mounted imaging and IoT sensors for pest and disease early warning systems are projected to reduce crop yield losses due to pests by an estimated 15%. These advancements demonstrate the transformative potential of AI and IoT technologies in modern agriculture, offering tangible benefits across various agricultural sectors.

In addition to these advancements, blockchain‐based full supply chain traceability systems have been successfully implemented for crops such as sugarcane, rice, wheat, and cotton, and others. These systems ensure transparency, security, and efficiency across processes like seedling propagation, field management, and harvesting. For example, the pilot implementation of blockchain technology at the national sugarcane seedling propagation base and the fully mechanized plantation demonstration base in Fusui County, Guangxi province has showcased its transformative potential. In the sugarcane pilot located at Fusui, blockchain‐H‐CPS achieved yield optimization (e.g., AI models trained on 10000+ soil samples reduced water usage by 25% via smart irrigation contracts), supply chain transparency (e.g., 100% traceability from planting Radio Frequency Identification (RFID) tagged seedlings to processing blockchain‐validated sugar batches). Farmers using the system reported an 18% cost reduction and 15% yield increase, demonstrating scalability for crops like rice and cotton.

This initiative not only enhanced production efficiency in sugarcane farming but also established a replicable and sustainable model for digital agricultural management on a national scale. Building on the success of these sugarcane‐focused projects, extending these solutions to other crops further underscores their adaptability and scalability. Insights gained from these pilots serve as a critical foundation for advancing China's agricultural sector toward precision agriculture, sustainability, and intelligent development.

Beyond pilot projects, research has also provided strong technical validation for blockchain‐based H‐CPS frameworks. For instance, a study proposed a blockchain‐based security framework for CPS and conducted simulation experiments, providing valuable insights into the integration of blockchain with H‐CPS. The experimental results demonstrated that integrating blockchain with CPS offers significant advantages in terms of performance and security. Using the Hyperledger Caliper tool, the integrated framework achieved strong transaction success rates and throughput under various workloads (100 to 1000 transactions per second), with a maximum throughput of 470 transactions per second and a success rate of 93%, while reducing latency to an average of 5 seconds. Compared to traditional Public Key Infrastructure (PKI) or Certificate Authority (CA) authentication methods, the certificateless mechanism of this framework reduced response times to 1 to 16 milliseconds, whereas traditional methods exhibited delays ranging from 40 to 242 milliseconds. Additionally, leveraging Practical Byzantine Fault Tolerance (PBFT) consensus protocols and distributed storage systems, the framework reduced energy consumption by approximately four times, decreased CPU usage by 50%, and demonstrated improved fault tolerance. These results validate the practical value of blockchain in enhancing the efficiency, security, and responsiveness of H‐CPS, while providing strong support for achieving distributed trust and transparency in agriculture.

## 6 Challenges and Prospects

### 6.1 Challenges

Despite its potential advantages, some issues still limit the practical deployment of blockchain‐empowered H‐CPS architecture for smart agriculture. While large‐scale agricultural equipment has become commercialized, factors such as agricultural scale, crop types, and labor costs may hinder the immediate commercialization of agrarian robots and drone clusters on farms. Therefore, further research is required to lower production thresholds and reduce procurement costs.^[^ ^117^ ^]^ Regarding small‐scale farms, training models using data from the physical system layer are insufficient, suggesting a need to incentivize data sharing to obtain more comprehensive training datasets.^[^ ^118^ ^]^ However, the training and inference computations heavily depend on communication and computing infrastructure. In rural areas, meeting the computational requirements may still be challenging, primarily because the network infrastructure development is relatively lagging, posing a challenge to deploying intelligent systems in agriculture.^[^ ^119^ ^]^ As a result, the research community is developing edge computing and cloud computing solutions to reduce extensive communication, demonstrating that hybrid edge‐cloud solutions are becoming increasingly feasible and cost‐effective in deploying smart agricultural systems.^[^ ^120^ ^]^ Fortunately, 5G and advanced wireless communication are other critical driving factors that improve rural networks and catalyze innovative agricultural systems development.

Introducing blockchain technology into smart agriculture also faces several challenges. One hand, its application in the supply chain requires broad participation and collaboration from all parties. However, reluctance from specific stakeholders to join the blockchain may arise, primarily due to the unfamiliarity or complexity of blockchain.^[^ ^121^ ^]^ Another reason is that some companies have already established supply chain management systems, and the incompatibility in a blockchain system may make them incur additional costs, discouraging a shift from existing operational methods,^[^ ^122^ ^]^ and hindering the blockchain. A key solution to this issue is to design user‐friendly, operationally simple, and highly compatible blockchain systems. Governments should also implement corresponding incentive measures and organize training sessions for relevant personnel on operating blockchain systems. On the other hand, inherent issues with blockchain can limit its application in the agricultural sector. As a result, scalability and data interoperability remain major challenges in consensus blockchain networks.^[^ ^123^ ^]^ The scalability challenge is particularly evident when considering the potential growth in the number of connected devices and data volumes in large‐scale agricultural environments. For instance, as farm sizes expand, the number of IoT devices generating data increases exponentially, which places significant demands on the computational and storage capacity of blockchain nodes.

To address the scalability challenge, we conducted related experiments and performed quantitative analyses.^[^ ^124^ ^]^ The results demonstrate the computational and storage efficiency of SemantiChain compared to traditional and state‐of‐the‐art retrieval systems. For instance, on the QUORA dataset,^[^ ^125^ ^]^ SemantiChain achieves a retrieval memory usage of only 10 MB, compared to 1532 MB (brute force), representing a reduction of over 99%. Similarly, average search time is reduced from 700.25 s (brute force) to 9.31 s, showcasing its suitability for handling large‐scale data in real‐world agricultural networks. Moreover, compared to the Merkle Semantic Trie DataBase (MSTDB),^[^ ^126^ ^]^ SemantiChain achieves at least 45.88% improvement in retrieval performance while reducing memory usage by 95.76%, further highlighting its scalability. These results suggest that blockchain can support large‐scale deployment in smart agriculture by balancing computational cost and retrieval efficiency, even in scenarios with increasing farm size or network complexity. Further optimization will still be necessary to tackle challenges associated with even larger‐scale deployments in the future.

Unfortunately, cross‐node communication and consensus processes may become bottlenecks, resulting in latency and reduced throughput in large and complex networks. These factors highlight that when blockchain provides decentralization and security benefits, its current architecture must be optimized to meet the demands of high‐throughput and large‐scale agricultural applications. Researchers and developers have explored various methods and technologies to improve blockchain systems' scalability, like layered structures, sharding techniques, and enhancements to consensus algorithms.^[^ ^127^ ^]^ Thus, the current methods in blockchain can only be stored indirectly through off‐chain databases for non‐text data such as images and audio.^[^ ^128^ ^]^ Some solutions involving integrating semantic features with blockchain have been introduced to address this issue.

### 6.2 Prospects

**Figure 6:**
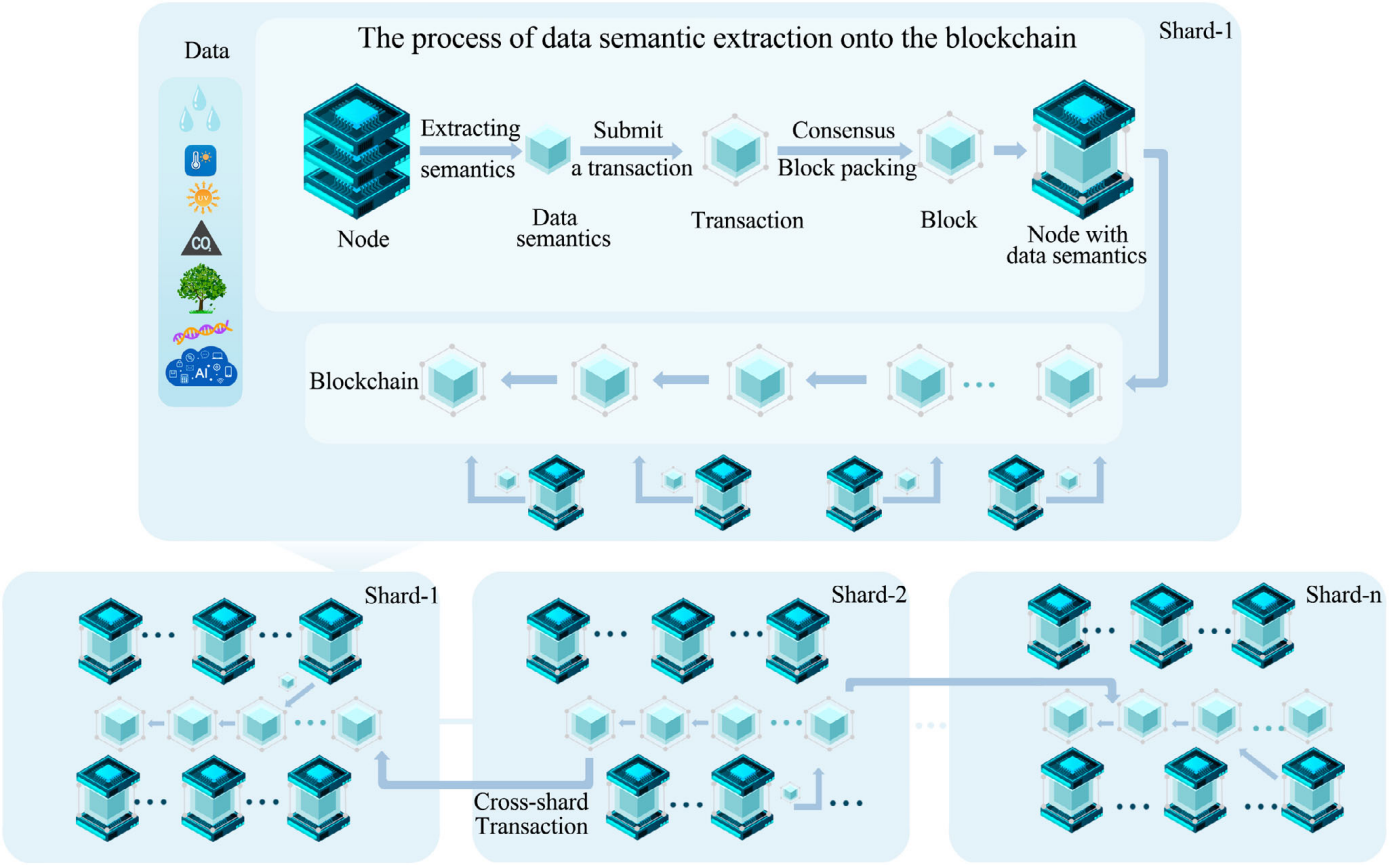
A semantic blockchain.

Blockchain sharding addresses the performance bottleneck caused by processing many transactions by dividing the entire blockchain network into small, independent shards. Each shard can process its transactions and smart contracts without waiting for confirmation from the whole network.^[^ ^129^ ^]^ This approach enhances parallelism, thereby increasing the throughput of the entire network. The semantic data model is an advanced representation of knowledge designed to capture the data attributes and relationships, allowing for a deeper understanding of each instance in the application context. By introducing sharding and semantic technologies, the scalability, intelligence, understandability, and interoperability of blockchain systems can be improved (**Figure** **6**). This method involves utilizing a series of steps, such as semantic extraction, transaction packaging, and consensus, to support cross‐regional agricultural data integration, precise execution of agricultural smart contracts, and the construction of farm knowledge and model graphs.^[^ ^130^ ^]^

**Figure 7:**
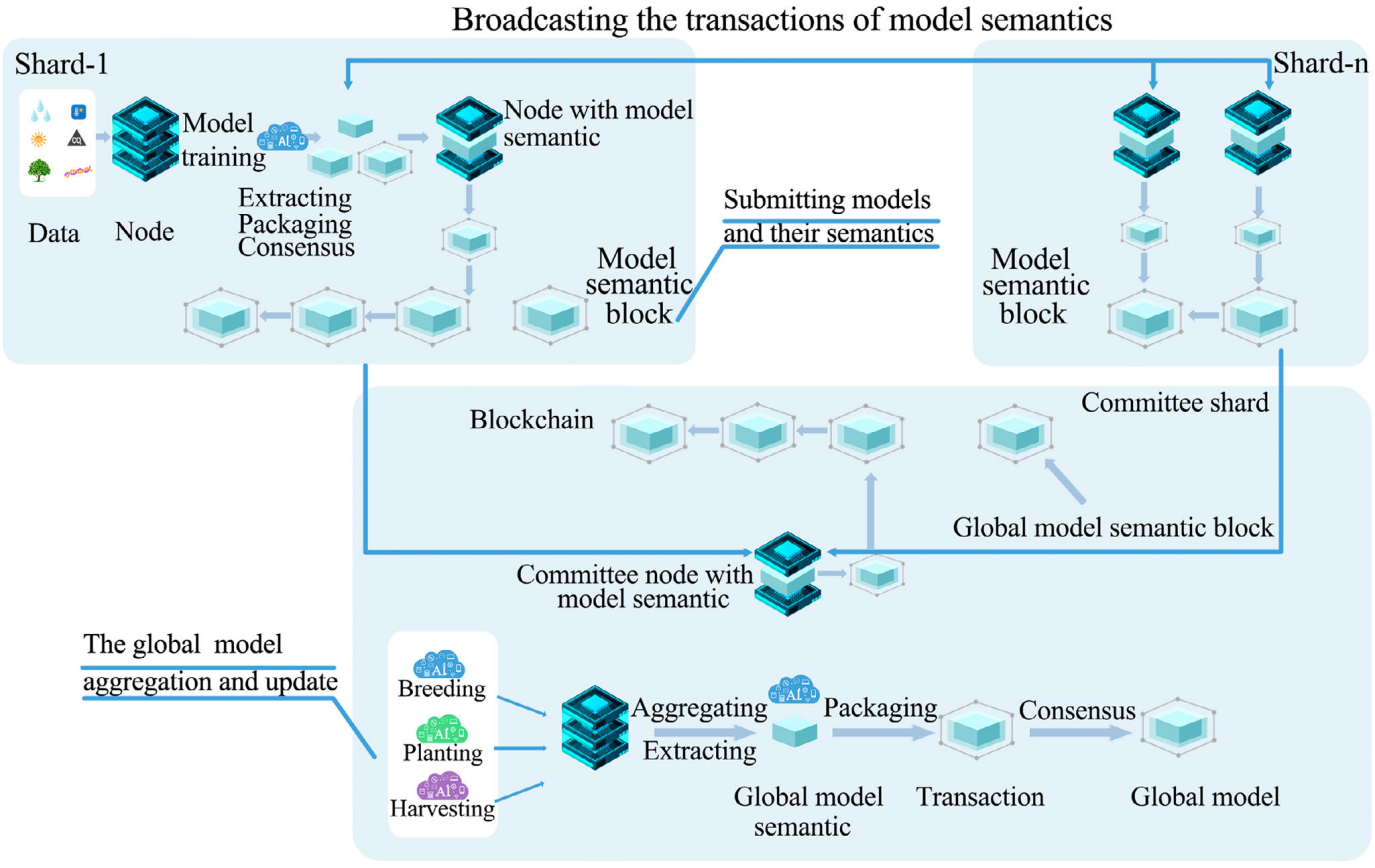
Federated learning on the shards of a semantic blockchain.

Integrating sharding and semantic technologies into the blockchain architecture is vital for smart agriculture for the following reasons: First, it helps to record the semantics of agricultural data transactions on the blockchain. For example, pedigree relationships of crops from different regions can be transformed into semantics and stored on dedicated blockchain shards, helping experts from various areas accurately query crop knowledge graphs on the blockchain for their respective regions to support subsequent breeding research. Second, this approach helps us to record the semantics of AI models in the form of blockchain transactions. For example, we can deploy federated learning on the blockchain through smart contracts, where this learning is used to train and integrate AI models for smart breeding, planting, and harvesting. Moreover, the semantics of these models are used to generate blockchain shards for the regions where the models operate (**Figure** **7**). This method reduces the space required for storing models on the blockchain, improving its scalability and efficiently querying different models on the blockchain. In this context, the semantic data model organizes multidimensional data such as crops, climate, and soil in a hierarchical manner by constructing ontologies and semantic trees. The leaf nodes represent specific agricultural data features (e.g., crop types or meteorological data), the intermediate nodes capture the relationships between these features (e.g., “affects soil fertility” or “determines yield”), and the root node integrates data from all shards into a global agricultural knowledge graph. This semantic‐based data management approach can be further enhanced with natural language processing techniques such as Bidirectional Encoder Representation from Transformers (BERT) embeddings,^[^ ^131^ ^]^ which encode metadata into high‐dimensional semantic vectors, enabling more efficient cross‐regional data retrieval. At the same time, the introduction of federated learning algorithms into blockchain systems facilitates distributed training through smart contracts. Shards can even be assigned model training tasks based on data similarity or geographic proximity, with lightweight consensus protocols used to aggregate updates. To reduce communication costs, gradient quantization or model scarification techniques are employed for inter‐shard data exchange. In addition, semantic hash functions are used to optimize the alignment of data and models across shards.^[^ ^124^ ^]^ Building on this foundation, the blockchain enables efficient interoperability of agricultural data and models. For instance, a query about optimal planting conditions can trigger cross‐shard collaborative computation through relay nodes, integrating soil data and climate models from different regions to return a comprehensive result. This mechanism can not only enhance the scalability of blockchain systems but also effectively support precise decision‐making and knowledge generation in smart agriculture.

## 7 Conclusion

This study integrates blockchain with emerging digital technologies like IoT, AI, and H‐CPS to enhance various aspects of agriculture. The innovation is expected to enhance innovation and sustainable agricultural production. H‐CPS architecture, tailored to the operational characteristics of agricultural systems, has been introduced into the agriculture sector. This architecture comprises a conceptual framework and critical enabling technologies built upon advanced technologies such as blockchain, drones, satellite remote sensing, and AI. The blockchain serves as the core of the entire architecture, a framework serving as a blockchain‐empowered H‐CPS architecture for intelligent agriculture. This architecture is designed in three layers: the physical system layer, the network system layer, and the human‐machine interaction layer. The physical system layer performs agricultural data collection using various deployed sensing tools. The network system layer stores the data and records on the blockchain using various regional blockchain nodes. This technology trains models using a federated learning approach, facilitating the integration of a universally deployable global model on the blockchain for all regions to assist human decision‐making. Moreover, the human‐machine interaction layer involves a visual and user‐friendly blockchain platform to view data, conduct transactions, and adjust devices in the physical system layer for tasks such as irrigation, fertilization, and mechanized harvesting. With the deployment of the blockchain‐empowered H‐CPS architecture, agricultural data/models have become more secure, trustworthy, and traceable. However, holding immense potential is vital to positively impact crop breeding, agricultural production decision‐making, agricultural product transactions, agrarian finance, product quality and safety, global food supply chain, and environmental sustainability.^[^ ^132^, ^133^ ^]^ In future research, we envision creating a novel semantic‐based blockchain framework that addresses the challenges of large volumes of data and massive artificial intelligence models in smart agriculture. By embedding semantic relationships and leveraging sharding techniques, this framework is expected to further enhance its ability to manage large datasets and complex models by improving data organization, retrieval efficiency, and interoperability. It will provide stronger support for precision agriculture and supply chain transparency while contributing to the broader goals of sustainable agriculture and global food security.

## Conflict of Interest

The authors declare no conflict of interest.

## Author Contributions

X.W. and Q.W. contributed equally to this work. X.W., H.Z., and X.Y. performed formal analysis, software, visualization, and wrote the original draft. Q.W. performed formal analysis, visualization, wrote the original draft. H.C. performed visualization, wrote the original draft, X.Y. performed visualization, and provided resources. M.J.P. performed visualization; wrote the original draft. M.L. performed conceptualization, project administration, wrote reviewed and edited. Y.Q. performed conceptualization, funding acquisition, project administration, resources, supervision, wrote reviewed and edited.
